# The regenerative rehabilitation collection: a forum for an emerging field

**DOI:** 10.1038/s41536-018-0058-z

**Published:** 2018-10-19

**Authors:** Fabrisia Ambrosio, Thomas A. Rando

**Affiliations:** 1 Department of Physical Medicine & Rehabilitation, University of Pittsburgh, Pittsburgh, PA 15213 USA; 2 McGowan Institute for Regenerative Medicine, Pittsburgh, PA 15219 USA; 3 Department of Bioengineering, University of Pittsburgh, Pittsburgh, PA 15213 USA; 4 Rehabilitation R&D REAP, VA Palo Alto Health Care System, Palo Alto, CA 94304 USA; 5 Glenn Center for the Biology of Aging, Stanford University School of Medicine, Stanford, CA 94305 USA; 6 Department of Neurology and Neurological Sciences, Stanford University School of Medicine, Stanford, CA 94305 USA

Increasingly, investigators from the fields of regenerative and rehabilitative medicine have been converging to create a new, cross-disciplinary field of “regenerative rehabilitation”. Regenerative rehabilitation has been defined as “the application of rehabilitation protocols and principles together with regenerative medicine therapeutics toward the goal of optimizing functional recovery through tissue regeneration, remodeling, or repair”.^1^ Increased communication and collaboration across these two fields is delivering promising new insights for the clinical application of regenerative medicine technologies. In recognition of this expanding interest, *npj Regenerative Medicine* is inaugurating a regenerative rehabilitation web collection, which will showcase state-of-the-art advances and considerations in the field.

Why is now the time to initiate a web collection on regenerative rehabilitation? Rehabilitative medicine has a foundation in evidence-based approaches that are designed to harness innate tissue regenerative capacity and optimize functional recovery after injury or in the setting of disease. These approaches span the application of mechanical, electrical and thermal stimuli to both promote the function of stem cells themselves and optimize the local microenvironment, or niche, in a way that is more favorable for stem cell function. Likewise, regenerative medicine is based on principles of cell and tissue biology to support the restoration of tissue structure and function. Though these two fields have historically progressed in parallel, growing evidence suggests that leveraging expertise and techniques across both of these domains has the potential to significantly enhance outcomes. This initial collection of manuscripts features two pre-clinical examples of successful regenerative rehabilitation paradigms, a meta- analysis of stem cell-based clinical studies in which rehabilitation is poised to play a crucial role, and a position statement from federal funding organizations which strongly support this line of research.

A number of studies over the past decades have investigated tissue engineering approaches for the treatment of severe muscle injuries. In the light of compelling evidence that mechanical stimuli play a critical role in dictating stem cell responses, bioreactors are commonly utilized as critical step for “priming” cells or engineered tissue constructs in vitro prior to transplantation. However, the effect of in vivo mechanical stimulation following cell transplantation has, until recently, been far less appreciated. Nakayama et al. demonstrate the ability of a three-dimensional nanofibrillar scaffold in combination with an exercise protocol to restore tissue structure and composition in a murine model of volumetric muscle loss (VML).^2^ In designing the construct, careful attention was taken to design the scaffold in a way that both is biocompatible and closely mimics the architecture and mechanical properties of native skeletal muscle. Despite the biomimetic nature of the construct, the investigators found that implantation of the scaffold alone yielded no benefit in vascularity or muscle regeneration. However, when mice were given access to voluntary running wheels one week following the VML injury, the regenerative response was markedly improved, as evidenced by an increased vascular density, number of mature neuromuscular junctions, and diameter of nascent myofibers.

The results of the studies by Nakayama et al are consistent with previous findings by Quarta et al. showing that the addition of treadmill running enhances functional outcomes following implantation of stem cell-seeded scaffolds into a mouse model of VML.^3^ In a follow up study, Quarta et al evaluate the effect of these stem cell-seeded scaffolds on whole muscle biomechanics following a VML injury.^4^ They found that there was no difference in the active and passive muscle mechanics of injured muscles and injured muscles implanted with a scaffold alone. On the other hand, implantation of stem cell-seeded scaffolds restored the active length- tension relationship and passive tension of VML-injured muscles to those resembling uninjured control counterparts. The finding that the twitch kinetics of injured muscles treated with cell- seeded scaffolds parallel those of uninjured muscles was particularly noteworthy, as these findings suggest that the donor-derived myofibers recapitulate the physiologic contractile characteristics of native, healthy tissue. In the light of these findings, it is perhaps not surprising that muscles treated with a stem cell-seeded scaffold were highly amenable to the beneficial effects of muscle loading by treadmill running.

As an increasing number of pre-clinical studies provide evidence in support of a regenerative rehabilitation paradigm to enhance outcomes, there is a need to better define how such strategies may be most effectively and efficiently tested in a clinical setting. Study design of a clinical trial to test a single intervention is often challenging owing to the need to recruit an adequate number of subjects, minimize the risk for bias, and identify primary outcome variables that will best identify the presence or absence of a treatment effect. When considering a combinatorial approach, such as the use of rehabilitation together with cellular therapeutics, the challenges increase substantially. Iijima and colleagues take an important first step towards better understanding the rigor with which the efficacy of cellular therapies are being tested in individuals with knee osteoarthritis (OA),^5^ a patient population that has consistently been shown to benefit from participation in rehabilitation programs.^6^ Meta-analysis of 35 studies investigating the transplantation of mesenchymal stem cells (MSCs) revealed not only that MSC implantation decreases OA pain levels, but that those studies that included a rehabilitation component were associated with improved self-reported physical functioning. The investigators raise the important point that none of the studies stratified patients according to the presence of a rehabilitation program, and they highlight the need for future studies to evaluate the potential synergy of physical therapeutics and MSC transplantation in the treatment of individuals with OA.

While the emergence of regenerative rehabilitation is exciting for its conceptual innovation and for its potential to significantly improve the efficacy of regenerative medicine therapeutics, there is a crucial need for commitment from funding agencies to foster these multidisciplinary collaborations in order for the field to grow. In a paper reflecting the perspectives of the Veterans Administration, the Department of Defense, the National Institutes of Health and the National Science Foundation, representatives from each institution converged to present a call for increased research in the field of regenerative rehabilitation.^7^ Each agency submitted a statement that outlines its respective interests in the field. The paper also summarizes key drivers that support the need for a paradigm shift in the way scientists and clinicians tackle the contemporary medical needs of civilian and military populations.

These articles exemplify the potential of regenerative rehabilitation to help accelerate advances in research and clinical practice for a broad array of pathologies, and they illustrate the continued progress toward the integration of regenerative medicine technologies with physical therapeutics. Furthermore, this collection highlights the ongoing commitment of *npj Regenerative Medicine* to serving as a venue for the publication of regenerative rehabilitation studies.
